# A (1;19) translocation involving *TCF3-PBX1* fusion within the context of a hyperdiploid karyotype in adult B-ALL: a case report and review of the literature

**DOI:** 10.1186/s40364-015-0029-0

**Published:** 2015-02-18

**Authors:** Carlos A Tirado, David Shabsovich, Lei Yeh, Sheeja T Pullarkat, Lynn Yang, Michael Kallen, Nagesh Rao

**Affiliations:** Department of Pathology and Laboratory Medicine, David Geffen UCLA School of Medicine, Los Angeles, CA 90024 USA

**Keywords:** *TCF3-PBX1*, hyperdiploidy, B-ALL, cytogenetics, FISH

## Abstract

**Background:**

The t(1;19)(q23;p13), which can result in the *TCF3-PBX1* chimeric gene, is one of the most frequent translocations in B-acute lymphoblastic leukemia (B-ALL) and is observed in both adult and pediatric populations at an overall frequency of 6%. It can occur in a balanced or unbalanced form and as a sole abnormality is associated with an intermediate prognosis. Additionally, this translocation is observed in the context of hyperdiploid B-ALL, in which case it is associated with a poor prognosis. However, due to different translocation partner genes at chromosomes 1 and 19, distinct subtypes of hyperdiploid B-ALL with t(1;19)/der(19)t(1;19) are recognized based on the presence or absence of the *TCF3-PBX1* fusion gene, but the cytogenetic and etiologic differences between the two remain understudied.

**Findings:**

We report a case of an adult with a history of relapsed precursor B-ALL whose conventional cytogenetics showed an abnormal female karyotype with both hyperdiploidy and a t(1;19)(q23;p13). Fluorescence in situ hybridization (FISH) on previously G-banded metaphases using the LSI TCF3/PBX1 Dual Color, Dual Fusion Translocation Probe confirmed the presence of the *TCF3-PBX1* gene fusion.

**Conclusions:**

This particular pattern with a *TCF3-PBX1* fusion within the context of a hyperdiploid karyotype is seen in B-ALL and is usually associated with a poor outcome. This case is one of only a few cases with both hyperdiploidy and a confirmed *TCF3*-*PBX1* fusion, demonstrating the importance of using FISH for proper molecular classification of these cases in order to distinguish them from those with hyperdiploidy but no *TCF3-PBX1* fusion gene. Such molecular studies may provide insight into the precise differences between *TCF3-PBX1* positive and negative hyperdiploid B-ALL bearing the t(1;19)(q23;p13).

## Introduction

The t(1;19)(q23;p13) is one of the most frequent translocations in B-acute lymphoblastic leukemia (B-ALL), and is observed in both adult and pediatric populations at an overall frequency of 6% . This translocation can occur in a balanced – t (1;19)(q23;p13) – or unbalanced – der(19)t(1;19)(q23;p13) – form and can result in the fusion of *TCF3* (transcription factor 3) found at 19p13 and *PBX1* (pre-B cell leukemia homebox 1) found at 1q23 to form a chimeric gene whose protein product alters cell differentiation arrest, among other cellular processes [1]. Specifically, the fusion gene encodes a transcription factor bearing the transactivation domain of *TCF3* and the DNA-binding domain of *PBX1*, which facilitates constitutive activation of genes bound by the protein encoded by *PBX1* and other PBX proteins [2]. As a sole abnormality, t(1;19)/der(19)t(1;19) is associated with an intermediate prognosis in B-ALL, and hyperdiploidy is associated with a favorable prognosis [1]. However, more rarely, cases of t(1;19)/der(19)t(1;19) within the context of a hyperdiploid karyotype have been observed, only some of which express the *TCF3-PBX1* fusion gene and are associated with a poor prognosis [3]. In addition to *PBX1*, other partner genes involved in rearrangements of *TCF3*, although at much lower frequencies, include *ZNF384* (12p13; prognosis unknown), *NOL1* (12p13; prognosis unknown), an unknown partner gene at 13q14 (prognosis unknown), *HLF* (17q22; extremely poor prognosis), and *FB1/TFPT* (19q13.4; prognosis unknown) [4-6]. The cytogenetic and etiologic differences between *TCF3-PBX1* positive and negative B-ALL with hyperdiploidy and t(1;19)/der(19)t(1;19) remain understudied due to lack of molecular classification of the cases reported in the literature.

## Case presentation

The patient was a forty-four year old woman with a history of relapsed precursor B-ALL, who was initially diagnosed in March 2013 with leukemic cells showing an immunophenotype positive for CD10, CD19, icCD22, CD38, icCD79a, CD138, TdT, HLA-DR and icIgM as well as a normal karyotype. Initial diagnosis was established at another institution at which point FISH analysis was not performed. After UK ALL 14 protocol consolidation therapy, she was considered to be in remission. In December 2013, a bone marrow biopsy showed evidence of relapse, and was comprised of approximately 85% blasts with a pre-B immunophenotype and a hyperdiploid, complex, poor-risk karyotype, further described in the results section. In January 2014, the patient underwent therapy with FLAG-Ida, resulting in a hypoplastic marrow with no significant residual blast population. Later in April 2014 she enrolled in a clinical trial with blinatumomab, which was eventually discontinued because the patient experienced multiple seizure episodes. A bone marrow biopsy showed extensive tumor necrosis with involvement by B-lymphoblasts representing over 90% of viable cells and comprising 5% of the total surface area. The immunophenotype was positive for CD10, CD19, PAX-5, CD79a and TdT (weak, rare), and negative for CD34 and CD20. The patient expired in May 2014 of relapsed B-lymphoblastic leukemia. Autopsy included a bone marrow biopsy, which revealed a hypercellular marrow of greater than 95% cellularity with sheets of lymphoblasts and extensive tumor necrosis.

## Material and methods

Chromosome analysis was performed using standard cytogenetic techniques on the bone marrow of this patient. The karyotypes were prepared using the Applied Imaging CytoVision software (Applied Imaging, Genetix, Santa Clara, CA) and described according to the ISCN 2013 nomenclature [7].

Fluorescence in situ hybridization (FISH) was performed on interphase nuclei using the Vysis MYC-IGH Dual Color, Dual Fusion Probe, Vysis LSI BCR,ABL ES Dual Color Translocation Probe, and Vysis LSI MLL Dual Color, Break Apart Rearrangement Probe from Abbott Molecular (Des Plaines, Illinois 60018). Additionally, FISH was performed with the LSI TCF3/PBX1 Dual Color, Dual Fusion Translocation Probe on previously G-banded metaphases.

## Findings

**Figure 1:**
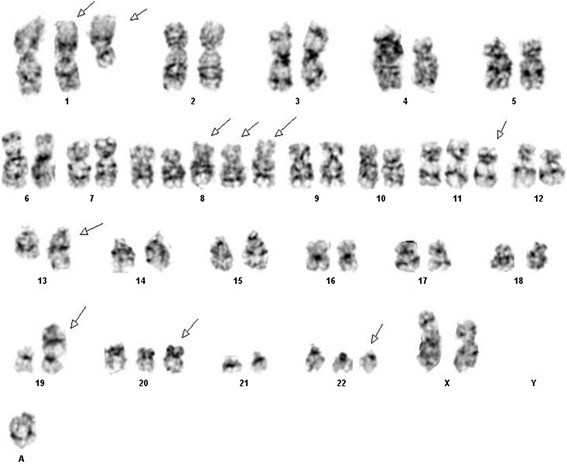
**Karyotype of female patient revealing t(1;19) in a hyperdiploid context.**

Only three metaphase cells were available for chromosome analysis due to a poor mitotic index. These cells revealed an abnormal female karyotype with numerical and structural abnormalities including extra copies of chromosomes 1, 8, 11, 20, 22, a (1;19) translocation, an unbalanced rearrangement of the long arm of chromosome 13 leading to 13q-, and a marker chromosome of unknown origin. This karyotype was described as (Figure 1):

$$ 53\hbox{--} 54,\mathrm{X}\mathrm{X},+1,\mathrm{t}\left(1;19\right)\left(\mathrm{q}23;\mathrm{p}13\right),+8,+8,+8,+11,\mathrm{add}(13)\left(\mathrm{q}34\right),+20,+22,+\mathrm{m}\mathrm{a}\mathrm{r}\left[\mathrm{c}\mathrm{p}3\right] $$

**Figure 2:**
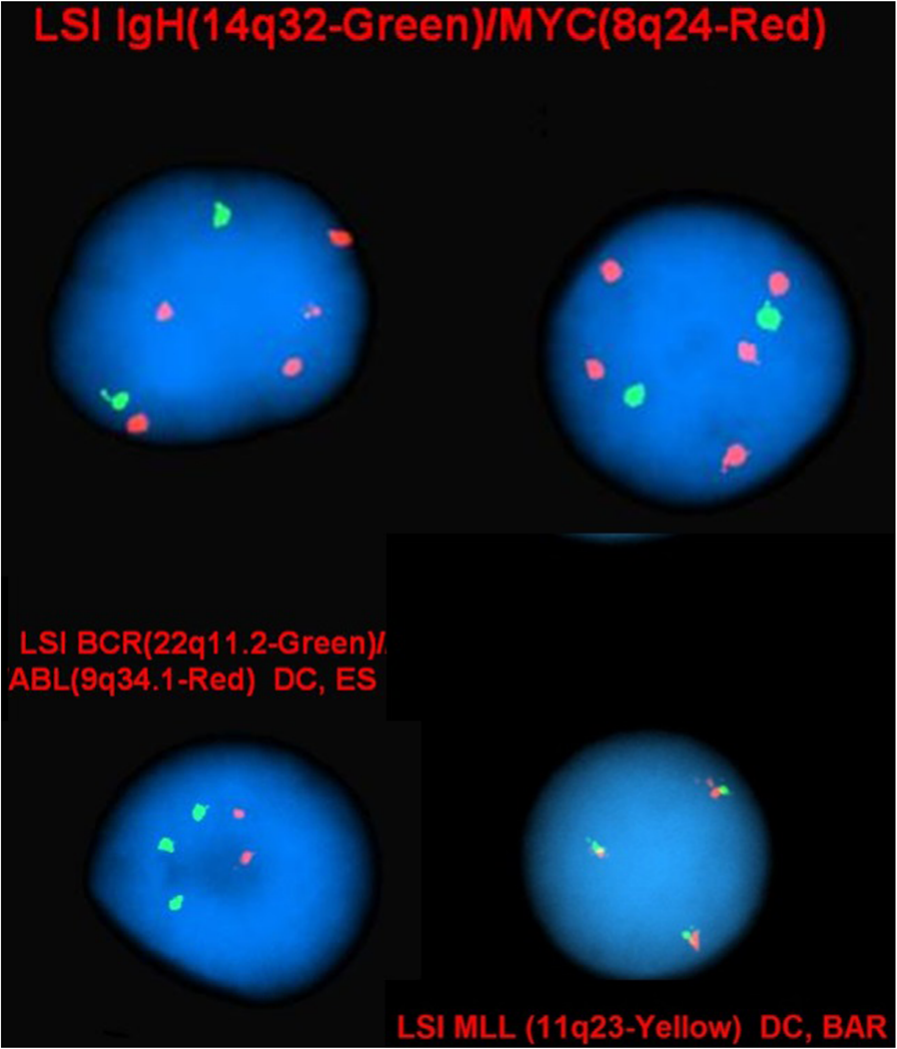
**FISH analysis was used to confirm the additional copies of chromosomes 8, 22 and 11.**

FISH on interphase nuclei confirmed the additional copies of chromosome 8 in 73.8% of nuclei (79/107), chromosome 22 in 80% of nuclei (44/55), as well as chromosome 11 in 4.7% of nuclei (4/85) examined. The FISH results (Figure 2) were described as:

- nuc ish(MYCx5,IGH)x2)[79/107]
- nuc ish(BCRx3,ABL1x2)[44/55]
- nuc ish(MLLx3)[4/85]

**Figure 3:**
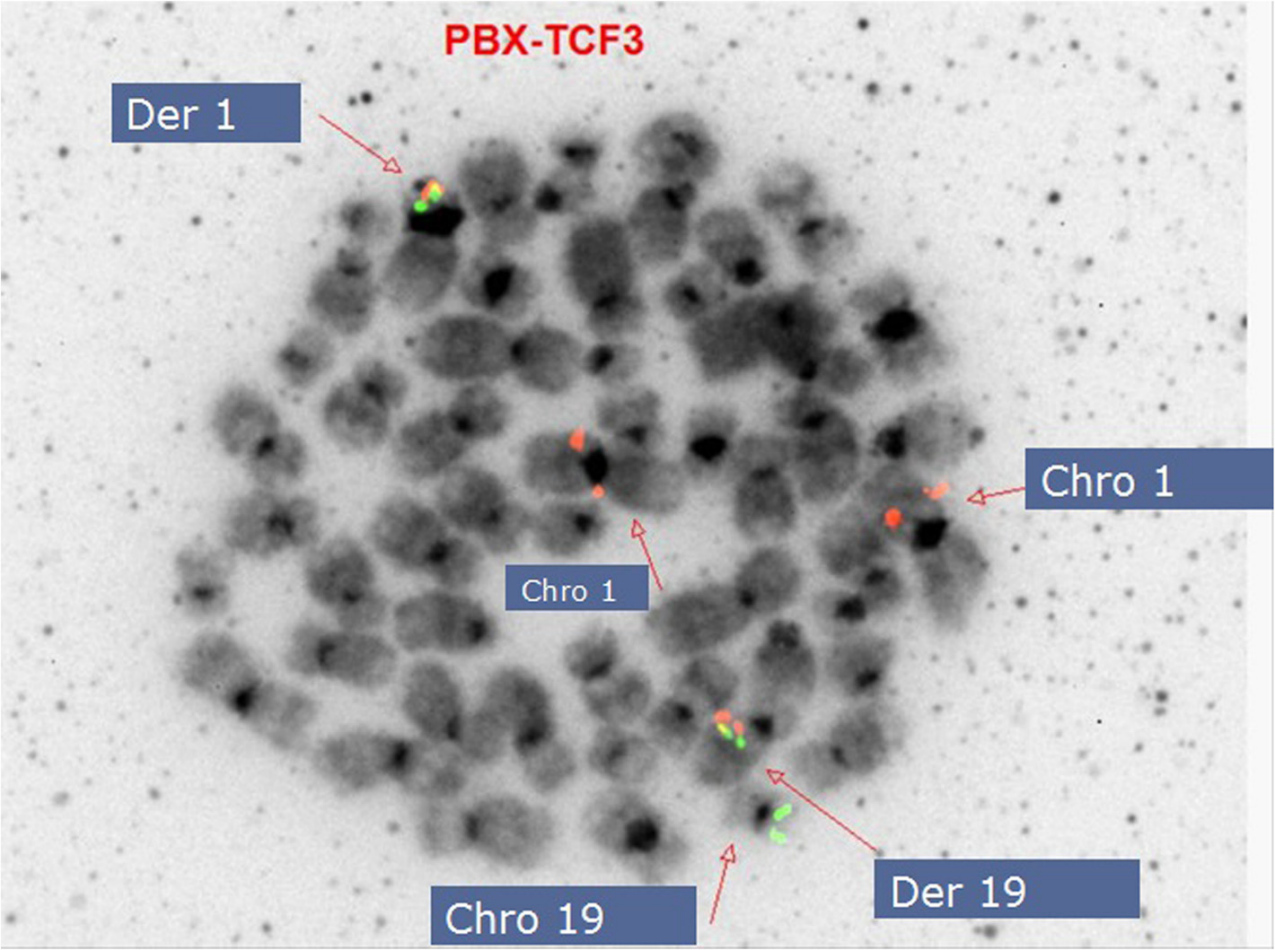
**FISH on a previously G-banded metaphase confirmed t(1;19)(q23;p13) involving the** ***TCF3*** **and** ***PBX1*** **genes, as well as an additional copy of chromosome 1.**

To further characterize and confirm the previous conventional cytogenetics findings [t(1;19) which fuses *TCF3* (green signal) on 19p13 with *PBX1* (red signal) at 1q23], FISH studies on previously G-banded metaphases were performed, and detected two fusion [t(1;19)] signals and an additional copy of red signal (+1q) with the *TCF3-PBX1* probe indicative of translocation between *TCF3* and *PBX1*, as well as an additional copy of the 1q23 locus, which is consistent with the karyotype results found previously. Gain of chromosome 1q is often seen in association with disease progression or advanced disease. Based on these studies the karyotype was described as (Figure 3):

$$ \begin{array}{l}53\hbox{-} 54,\mathrm{X}\mathrm{X},+1,\mathrm{t}\left(1;19\right)\left(\mathrm{q}23;\mathrm{p}13\right),+8,+8,+8,+11,\mathrm{add}(13)\left(\mathrm{q}34\right),+20,+22,+\mathrm{m}\mathrm{a}\mathrm{r}\left[\mathrm{c}\mathrm{p}3\right].\mathrm{i}\mathrm{s}\mathrm{h}\\ {}\left(\mathrm{P}\mathrm{B}\mathrm{X}1\mathrm{x}4\right)\left(\mathrm{T}\mathrm{C}\mathrm{F}3\mathrm{x}3\right)\left(\mathrm{P}\mathrm{B}\mathrm{X}1\kern0.5em \mathrm{con}\kern0.5em \mathrm{T}\mathrm{C}\mathrm{F}3\mathrm{x}2\right)\end{array} $$

## Discussion

The t(1;19)(q23;p13)/der(19)t(1;19)(q23;p13) is one of the most common translocations seen in B-ALL cases and is typically found as a sole abnormality. It creates a fusion of *TCF3* on 19p13 with *PBX1* at 1q23, can be present in balanced or unbalanced form, and is usually associated with an intermediate prognosis [1]. Hunger et al. noted in an early study that 95% of t(1;19)/der(19)t(1;19)-positive cases of B-ALL with <50 chromosomes expressed the *TCF3-PBX1* fusion transcript, whereas only 25% of cases with >50 chromosomes did. Furthermore, immunophenotypic differences between *TCF3-PBX1* positive and *TCF3-PBX1* negative cases were observed, which suggested etiologic differences between the two subtypes [3].

In a recent study conducted by Paulsson et al., 42 cases with both t(1;19)/der(19)t(1;19) and high hyperdiploidy (HeH; 51–67 chromosomes) from both published literature and the LRCG database were analyzed, revealing similar numerical chromosomal gains in both translocation-HeH (t-HeH) and classic-HeH (c-HeH) cases, most commonly involving chromosomes 21, 4, 6, 10, 18, 14, X, and 17, in decreasing frequency [8]. Furthermore, none of these cases were found to have a stemline balanced or unbalanced t(1;19), whereas 11% had hyperdiploid stemlines, suggesting that numerical chromosomal gains resulting in HeH are primary cytogenetic aberrations and occur prior to t(1;19)/der(19)t(1;19) [8]. This may result in clinical similarities between t(1;19)/der(19)t(1;19)-HeH ALL and c-HeH ALL, as the two may share a similar cytogenetic progression and etiology. It was also found that the majority of t(1;19)/der(19)t(1;19)-HeH cases tested by molecular methods were negative for the presence of the *TCF3-PBX1* fusion gene [8]. Additionally, previous studies have found that greater than 90% of t(1;19) positive, *TCF3-PBX1* fusion negative cases have an unbalanced form of the rearrangement [4]. In Paulsson et al’s study, only 18% of the cases had a balanced rearrangement [8], while 40% of *TCF3-PBX1* positive cases overall have been found to have a balanced rearrangement [1], ultimately suggesting etiologically distinct subtypes of B-ALL with both hyperdiploidy and t(1;19)/der(19)t(1;19) based on the presence of the *TCF3-PBX1* fusion gene by FISH and/or polymerase chain reaction (PCR) [8].

In the present study, we report a case of hyperdiploid B-ALL with a balanced t(1;19) bearing the *TCF3-PBX1* fusion gene confirmed by metaphase FISH, which has only been previously reported in a small number of cases and represents a distinct subtype of B-ALL based on the presence of the confirmed fusion gene in conjunction with hyperdiploidy and t(1;19)/der(19)t(1;19). Interestingly, the numerical gains present in our case, of chromosomes 1, 8, 11, 20, and 22, are not consistent with the most common gains found in t-HeH/t(1;19)-positive B-ALL by Paulsson et al. [8]. In that study, the majority of cases were found to have unbalanced rearrangements and out of those that had molecular evidence, most did not bear the *TCF3-PBX1* fusion [8]. The presence of different numerical gains between our case and those of Paulsson et al. further supports the fact that the *TCF3-PBX1* positive and negative variants of t(1;19)/hyperdiploid B-ALL represent distinct subtypes of the disease. Furthermore, we queried the Mitelman Database of Chromosome Aberrations in Cancer for reported cases of ALL with t(1;19)/der(19)t(1;19), hyperdiploidy, and molecular evidence (FISH and/or PCR) of *TCF3-PBX1* fusion, and only identified 5 cases that were positive for the fusion (Table 1). When compiling the karyotypes of these cases and the present case, we noted that four out of six cases had additional copies of chromosome 8, which was interestingly not found to be one of the most common numerical gains in t-HeH/t(1;19)-positive B-ALL [8].

**Table 1:** **Cases of adult B-ALL with hyperdiploidy, t(1;19)/der(19)t(1;19), and** ***TCF3-PBX1*** **fusion confirmed by FISH and/or polymerase chain reaction (PCR)**

**Case**	**Age/Sex**	**Karyotype**	**Reference**
1***	22/F	47,XX,t(1;19),+8/46,XX,-19,+der(19)t(1;19)	[2]
2*	51/M	47,XY,add(1)(p36),der(3)(t3;?)(q23;?),+7,t(14;18)(q32;q21),t(15;22)(q26;q21),der(19)t(1;19)(q23;p13)[26]/46,XY[14]	[13]
3*	38/M	56,XY,+4,+5,+6,+7,+8,+10,?del(10)(q22q24),+16,+19,der(19)t(1;19)(q23;p13),+21,der(21)t(1;21)(q11;p11.1),+22,inc[4]/57,idem,+14[4]/56,idem,-Y,+14[4]	[3]
4***	23/F	51,XX,+X,+5,+8,add(7)(p?),der(19)t(1;19)(q23;p13),+21,+22[13]/52,XX,+X,+5,+8,add(7)(p?),der(19)t(1;19)(q23;p13),+21,+22,+mar[2]	[14]
5**	21/M	48,XXY?c,t(1;19)(q23;p13),del(1)(p22),+mar[4]/47,XXY?c[9]	[14]
6***	44/F	53-54,XX,+1,t(1;19)(p23;q13),+8,+8,+8,+11,add(7)(q34),+20,+22,+mar[cp3].ish(PBX1x4)(TCF3x3)(PBX1 con TCF3 x2)	This report

Prognostic data were limited for these cases.

*Reference does not provide further clinical/prognostic data about the malignancy.

**Reference indicates that disease did not relapse.

***Reference indicates that disease relapsed.

Recent molecular insights into the *TCF3-PBX1* fusion protein have revealed its involvement in complex signaling pathways. In particular, deregulation of JunD and NFX1-regulated transcriptional processes has been noted to be a significant effect of the fusion protein [9]. Additionally, *PAX5* (19p13.2) haploinsufficiency, detectable both by conventional and molecular cytogenetics, is associated with *TCF3-PBX1* in B-ALL. Specifically, FISH using both *TCF3* split signal probes in conjunction with *PAX5* locus-specific deletion probes suggests that *PAX5* is a secondary event in the oncogenesis of *TCF3-PBX1*-positive B-ALL, and may be associated with clonal evolution of the malignancy [10]. Furthermore, studies have revealed that vascular endothelial growth factor-C (VEGF-C), encoded by *VEGFC* (4q34.3), is involved and perhaps essential to proliferation of *TCF3-PBX1* positive leukemic B cells [11]. Finally, treatment with hyperfractionated cyclophosphamide, vincristine, doxorubicin, and dexamethasone alternating with methotrexate and high-dose cytarabine (hyper-CVAD) has shown a favorable outcome in adults with t(1;19)-positive ALL [12].

Hyperdiploidy in B-ALL normally conveys a favorable prognosis, but in the present study, the particular pattern of a t(1;19)(q23;p13.3) with *TCF3-PBX1* fusion within the context of a complex karyotype (>3 abnormalities) and hyperdiploidy due to extra copies of chromosomes 8, 11 and 22 (confirmed by FISH) plus the presence of a marker chromosome of unknown origin is associated with an unfavorable prognosis in B-ALL [3]. It is one of only a few published cases with hyperdiploidy, t(1;19)/der(19)t(1;19), and a confirmed *TCF3-PBX1* fusion in B-ALL, demonstrating the importance of using FISH and PCR for proper cytogenetic and molecular classification in order to distinguish the present scenario from hyperdiploid B-ALL with t(1;19)/der(19)t(1;19), but lacking the *TCF3-PBX1* fusion. The latter represents a different subtype of B-ALL that may be primarily driven by chromosomal gains or other fusion genes rather than the t(1;19)/der(19)t(1;19) resulting in the *TCF3-PBX1* fusion and should not be confused with the entity presented in this report. Further investigation of the cytogenetic and molecular etiologies of these subtypes of B-ALL is warranted to determine their implications in the diagnosis and prognosis of the malignancy.
